# Perioperative management and anaesthetic considerations in pelvic exenterations using Delphi methodology: results from the PelvEx Collaborative

**DOI:** 10.1093/bjsopen/zraa055

**Published:** 2021-02-15

**Authors:** PelvEx Collaborative, A Y Chok, A Oliver, S Rasheed, E J Tan, M E Kelly, A G J Aalbers, N Abdul Aziz, N Abecasis, M Abraham-Nordling, T Akiyoshi, W Alberda, M Albert, M Andric, E Angenete, A Antoniou, R Auer, K K Austin, O Aziz, R P Baker, M Bali, G Baseckas, B Bebington, M Bedford, B K Bednarski, G L Beets, P L Berg, J Beynon, S Biondo, K Boyle, L Bordeianou, A B Bremers, M Brunner, P Buchwald, A Bui, A Burgess, J W A Burger, D Burling, E Burns, N Campain, S Carvalhal, L Castro, A Caycedo-Marulanda, K K L Chan, G J Chang, M H Chew, P Chong, H K Christensen, H Clouston, M Codd, D Collins, A J Colquhoun, A Corr, M Coscia, P E Coyne, B Creavin, R S Croner, L Damjanovic, I R Daniels, M Davies, R J Davies, C P Delaney, J H W de Wilt, Q Denost, C Deutsch, D Dietz, S Domingo, E J Dozois, M Duff, T Eglinton, J M Enrique-Navascues, E Espin-Basany, M D Evans, N S Fearnhead, K Flatmark, F Fleming, F A Frizelle, M A Gallego, E Garcia-Granero, J L Garcia-Sabrido, L Gentilini, M L George, V George, L Ghouti, F Giner, N Ginther, R Glynn, T Golda, B Griffiths, D A Harris, J A W Hagemans, V Hanchanale, D P Harji, R M Helewa, G Hellawell, A G Heriot, D Hochman, W Hohenberger, T Holm, A Holmström, R Hompes, J T Jenkins, S Kaffenberger, G V Kandaswamy, S Kapur, Y Kanemitsu, S R Kelley, D S Keller, M S Khan, H Kim, H J Kim, C E Koh, N F M Kok, R Kokelaar, C Kontovounisios, H Ø Kristensen, H M Kroon, M Kusters, V Lago, S G Larsen, D W Larson, W L Law, S Laurberg, P J Lee, M Limbert, M L Lydrup, A Lyons, A C Lynch, C Mantyh, K L Mathis, C F S Margues, A Martling, W J H J Meijerink, S Merkel, A M Mehta, D R McArthur, F D McDermott, J S McGrath, S Malde, A Mirnezami, J R T Monson, J R Morton, T G Mullaney, I Negoi, J W M Neto, B Nguyen, M B Nielsen, G A P Nieuwenhuijzen, P J Nilsson, S T O’Dwyer, G Palmer, E Pappou, J Park, D Patsouras, G Pellino, A C Peterson, G Poggioli, D Proud, M Quinn, A Quyn, R W Radwan, P C Rasmussen, E Rausa, S E Regenbogen, A Renehan, R Rocha, M Rochester, J Rohila, J Rothbarth, M Rottoli, C Roxburgh, H J T Rutten, É J Ryan, B Safar, P M Sagar, A Sahai, A Saklani, T Sammour, R Sayyed, A M P Schizas, E Schwarzkopf, V Scripcariu, C Selvasekar, I Shaikh, D Shida, A Simpson, N J Smart, P Smart, J J Smith, A M Solbakken, M J Solomon, M M Sørensen, S R Steele, D Steffens, K Stitzenberg, L Stocchi, N A Stylianides, T Swartling, H Sumrien, P A Sutton, T Swartking, C Taylor, J Teras, R Thurairaja, E L Toh, P Tsarkov, Y Tsukada, S Tsukamoto, J J Tuech, W H Turner, J B Tuynman, G H van Ramshorst, D van Zoggel, W Vasquez-Jimenez, C Verhoef, G Vizzielli, E L K Voogt, K Uehara, C Wakeman, S Warrier, H H Wasmuth, K Weber, M R Weiser, J M D Wheeler, J Wild, M Wilson, A Wolthuis, H Yano, B Yip, J Yip, R N Yoo, D C Winter, P P Tekkis

## Abstract

**Background:**

The multidisciplinary perioperative and anaesthetic management of patients undergoing pelvic exenteration is essential for good surgical outcomes. No clear guidelines have been established, and there is wide variation in clinical practice internationally. This consensus statement consolidates clinical experience and best practice collectively, and systematically addresses key domains in the perioperative and anaesthetic management.

**Methods:**

The modified Delphi methodology was used to achieve consensus from the PelvEx Collaborative. The process included one round of online questionnaire involving controlled feedback and structured participant response, two rounds of editing, and one round of web-based voting. It was held from December 2019 to February 2020. Consensus was defined as more than 80 per cent agreement, whereas less than 80 per cent agreement indicated low consensus.

**Results:**

The final consensus document contained 47 voted statements, across six key domains of perioperative and anaesthetic management in pelvic exenteration, comprising preoperative assessment and preparation, anaesthetic considerations, perioperative management, anticipating possible massive haemorrhage, stress response and postoperative critical care, and pain management. Consensus recommendations were developed, based on consensus agreement achieved on 34 statements.

**Conclusion:**

The perioperative and anaesthetic management of patients undergoing pelvic exenteration is best accomplished by a dedicated multidisciplinary team with relevant domain expertise in the setting of a specialized tertiary unit. This consensus statement has addressed key domains within the framework of current perioperative and anaesthetic management among patients undergoing pelvic exenteration, with an international perspective, to guide clinical practice, and has outlined areas for future clinical research.

## Teaser

The PelvEx Collaborative consensus statement systematically addresses the perioperative and anaesthetic management of patients undergoing pelvic exenteration (PE). Using the modified Delphi methodology, recommendations across six key clinical domains comprising preoperative assessment and preparation, anaesthetic considerations, perioperative management, anticipating possible massive haemorrhage, stress response and postoperative critical care, and pain management were developed.

pelvic exenteratio and recommendation

## Resumen

**Antecedentes:**

El manejo perioperatorio y anestésico multidisciplinario de los pacientes sometidos a una exenteración pélvica (EP) es esencial para obtener buenos resultados quirúrgicos. No se han establecido pautas claras y existe una amplia variación en la práctica clínica a nivel internacional. Esta declaración de consenso consolida la experiencia clínica y las mejores prácticas de manera colectiva, y aborda sistemáticamente los dominios clave en el manejo perioperatorio y anestésico.

**Métodos:**

Se utilizó la metodología Delphi modificada para lograr el consenso en el grupo *PelvEx Collaborative*. El proceso incluyó una ronda de cuestionario en línea (*online)* que incluía comentarios controlados y una respuesta estructurada de los participantes, dos rondas de edición y una ronda de votación en línea. Se llevó a cabo de diciembre de 2019 a febrero de 2020. El consenso se definió como un acuerdo de más del 80%; menos del 80% de acuerdo indicó un bajo consenso.

**Resultados:**

El documento de consenso final contenía 47 declaraciones votadas, en seis dominios clave del manejo perioperatorio y anestésico en las EP, que comprendían evaluación y preparación preoperatorias, consideraciones anestésicas, manejo perioperatorio, anticipación de posibles hemorragias masivas, respuesta al estrés y cuidados críticos posoperatorios y manejo del dolor. Las recomendaciones de consenso se desarrollaron sobre la base del acuerdo de consenso alcanzado en 34 declaraciones.

**Conclusión:**

El manejo perioperatorio y anestésico de los pacientes con EP mejora mediante un equipo multidisciplinario dedicado con experiencia en los dominios relevantes en el marco de una unidad terciaria especializada. Bajo una perspectiva internacional, esta declaración de consenso ha abordado dominios clave del marco del actual del manejo perioperatorio y anestésico de pacientes con EP como guía de la práctica clínica y ha definido áreas para la investigación clínica futura.

## Introduction

Perioperative and anaesthetic management undertaken for pelvic exenteration requires close multidisciplinary and interprofessional collaboration between the anaesthetic and surgical teams, nursing staff and connected health members. These cases can be complex and heterogeneous in terms of patient profile, tumour characteristics and specific anatomical considerations, risk of bleeding, anticipated operating time, and type of exenterative and reconstructive procedure required. There have been no clear guidelines established for the optimal perioperative and anaesthetic management of these patients, with wide variations across different centres internationally.

Through this consensus statement, this international collaborative group sought to consolidate clinical experience and best practice collectively, based on current standards of care at this point in time. The objective was to provide recommendations and streamline perioperative strategies to improve surgical outcomes among patients undergoing pelvic exenteration. The PelvEx Collaborative has worldwide representation from specialist centres across five different continents, established with the central aim of ascertaining factors and practices associated with improved outcomes after pelvic exenterative surgery. In addition, the purpose of the consensus was to support and supplement perioperative clinical management by healthcare teams involved in treating patients undergoing pelvic exenterative.

The present consensus has been developed based on the following six perioperative clinical domains: preoperative assessment and preparation; anaesthetic considerations; perioperative management; anticipating possible massive haemorrhage; stress response and postoperative critical care; and pain management. Although the consensus recommendations can guide surgical and anaesthetic teams in their clinical practice, there is a need to individualize the care of each patient undergoing pelvic exenteration within the context of the prevailing clinical scenario. This consensus is not meant to supersede clinical judgment, but to provide a framework to guide decision-making.

## Methods

The modified Delphi methodology was used to achieve consensus from the PelvEx Collaborative, an iterative process adopted to gather consensus from specialist participants about issues within a predetermined scope of clinical research, where expert opinion is important in the absence of high-level definitive evidence^1^^,^^2^. Held from December 2019 to February 2020, the significant issues, challenges and considerations pertaining to perioperative care and anaesthesia specific to patients undergoing pelvic exenteration were defined by the pelvic exenterative team at the Royal Marsden Hospital. The Consensus Statement from the PelvEx Collaborative, an international group comprising surgeons, anaesthetists, radiologists, specialist nurses, and physicians including medical oncologists and radiation oncologists, is representative of the multidisciplinary team (MDT) approach necessary in the perioperative management of patients undergoing pelvic exenteration.

The consensus process included one round of online questionnaire involving controlled feedback and structured participant response, two rounds of editing, and one round of web-based voting. The study was performed using the commercially available online platform accessible through Typeform (Barcelona, Spain).

In round 1, there were 45 statements including three open-ended questions; the feedback and issues raised formed the basis of consensus statements for the subsequent round. In round 2, 28 key statements were fielded to the same group of participants. For each statement, consensus was achieved with more than 80 per cent agreement; less than 80 per cent agreement indicated low consensus. Owing to the conclusive responses obtained after two questionnaire rounds, further rounds were deemed unnecessary.

Each statement was graded by strength of recommendation and level of evidence according to the following two predefined scales, outlined in *Tables 1* and *2*,^3^. The medical evidence was critically evaluated for quality, including internal and external validity for the patients undergoing pelvic exenteration. RCTs and meta-analyses of RCTs were regarded as the highest level of evidence. In the absence of high-quality evidence, consensus of expert opinion was obtained on the relevant key aspects in the perioperative management of patients undergoing pelvic exenteration.

**Table 1:** Classes of recommendations

Class of recommendation	Definition	Suggested wording
I	Evidence and/or general agreement that a given treatment or procedure is beneficial, useful and effective	Is recommended/is indicated
II	Conflicting evidence and/or a divergence of opinion about the usefulness/efficacy of the given treatment or procedure	
IIa	Weight of evidence/opinion is in favour of usefulness/efficacy	Should be considered
IIb	Usefulness/efficacy is less well established by evidence/opinion	May be considered
III	Evidence/general agreement that the given treatment/procedure is not useful/ effective and may sometimes be harmful	Is not recommended

**Table 2:** Levels of evidence

Level of evidence	Description
A	Data derived from multiple RCTs or meta-analyses
B	Data derived from a single RCT or large non-randomized studies
C	Consensus of expert opinion and/or small studies, retrospective studies, registries

**Table 3:** Summary of consensus statements

Section	Recommendations
Preoperative assessment and preparation	Anaesthetic preassessment of high-risk pelvic exenteration Perform cardiopulmonary exercise testing If more than two cardiac risk factors and four or fewer METs, perform imaging stress testing before pelvic exenteration Formal assessment by a cardiologist is not required routinely
Anaesthetic considerations	Arterial line placement Consider large-bore vascular catheter Monitor arterial blood gases and haemoglobin regularly Keep plasma lactate level below 1 mmol/l, or as low as possible For high-risk pelvic exenteration procedures with expected duration longer than 12–18 h, another anaesthetic colleague should assist Dedicated anaesthesia team of nurses or operating department practitioners
Perioperative management	Routine MDT briefing before induction of anaesthesia Mechanical calf compression and TED stockings Pressure-point protection padding Careful leg positioning to avoid leg compartment syndrome; lower legs every 2–4 h Prone position may be required for surgical access or myocutaneous flap reconstruction
Anticipating possible massive haemorrhage	Cross-match at least 2 units of blood Tranexamic acid if required Consider thromboelastography in major haemorrhage Blood products ready for immediate use if required
Stress response and postoperative critical care	Noradrenaline as initial inotrope Transfer to CCU for stabilization before extubation if patient develops major SIRS or instability on inotropes CCU admission for invasive monitoring and/or organ support Goal-directed fluid therapy
Pain management	Regional techniques (such as epidural) can reduce postoperative opioid use Careful postoperative pain management protocol to maintain good regional anaesthesia

MET, metabolic equivalent of tasks; MDT, multidisciplinary team; TED, thromboembolic deterrent; CCU, critical care unit.

The PelvEx Collaborative consensus statement systematically addresses the perioperative and anaesthetic management of patients undergoing pelvic exenteration. Using the modified Delphi methodology, recommendations across six key clinical domains were developed, comprising preoperative assessment and preparation, anaesthetic considerations, perioperative management, anticipating possible massive haemorrhage, stress response and postoperative critical care, and pain management.

## Results

### Section 1: Preoperative assessment and preparation

For patients requiring pelvic exenteration, the aims of preoperative assessment should be determining the level of surgical fitness, ascertaining functional reserve and the ability to withstand periods of significant physiological stress, and the identification of issues that can potentially result in adverse surgical and anaesthetic outcomes. As patients undergoing pelvic exenteration comprise a heterogeneous group with diverse individual patient profiles, medical co-morbidities and varying levels of performance status, there has been no consensus on their optimal risk stratification.

Given the inherent risk involved in this surgical procedure, appropriate anaesthetist-led assessment and management of various co-morbid conditions in the perioperative period can considerably improve patient safety, especially among high-risk patients^4^. Generally, these high-risk patients have medical conditions that may include, but are not limited to, ischaemic heart disease, heart failure, valvular heart disease, cardiac implantable electronic device, pulmonary disease, and diabetes mellitus. The increased risk of perioperative death is stratified based on the aggregate of factors, including age, medical history, physiological and nutritional parameters, and current performance status. Although the ‘high-risk’ patient remains poorly defined, it has been suggested that patients with a mortality risk greater than 5 per cent, and those undergoing a procedure carrying a mortality risk of more than 5 per cent, be defined as high-risk surgical patients^5^.

Over the years, the role of the anaesthetist has broadened, to incorporate assessing the risk of anaesthesia, optimizing co-morbid conditions if possible, selecting patients who may benefit from additional regional anaesthetic modalities, and identifying potential difficulties that may be encountered during the operation and subsequent postoperative course.

Cardiopulmonary exercise testing (CPET) provides an integrated objective measure of aerobic fitness and functional capacity, which in turn is a strong predictor of postoperative morbidity and mortality^6^. It is also useful to detect unexpected co-morbidity, thereby guiding further testing if necessary and allowing possible optimization before surgery^7^. Ascertaining functional capacity is a vital step in preoperative risk assessment and is measured in metabolic equivalent of tasks (METs). While 1 MET represents basal metabolic rate, the inability to climb two flights of stairs or run a short distance (4 or fewer METs) suggests poor functional capacity and is therefore associated with adverse perioperative outcomes, particularly cardiac events^8^.

Cardiovascular complications are one of the leading causes of postoperative morbidity and mortality^9^. The most validated risk-scoring system, the Revised Cardiac Risk Index, which consists of one procedural and five clinical risk factors^10^, has been shown to have a linear relationship with the likelihood of perioperative cardiac complications^11^. In a well and fit patient, routine cardiological assessment is unnecessary on the premise of cost-effective healthcare, due to the relatively low yield of additional investigations that are therefore unlikely to alter clinical management. However, in the patient with more than two clinical risk factors and poor functional capacity (4 or fewer METs), the value of imaging stress-testing and myocardial perfusion imaging before pelvic exenteration is well established^8^, as important prognostic information can be derived based on the extent of ischaemic myocardium under stress.

With differing logistical considerations and arrangements in the preoperative preparation across the numerous specialized units worldwide, there was no consensus on whether patients requiring pelvic exenteration should be admitted routinely the night before surgery.

#### Consensus recommendations

1. *If allocated time and resources, the anaesthetist undertaking the case should personally preassess the high-risk patient requiring pelvic exenteration, to help prepare for forthcoming surgery.* Class I/level C (84 per cent)
2. *CPET, if available, is useful for assessing the patient's functional capacity before proceeding with pelvic exenteration.* Class I/level B (86 per cent)
3. *At preassessment, if the patient has had a very good CPET result and is deemed low risk for major perioperative morbidity, pelvic exenteration can be undertaken.* Class I/level B (100 per cent)
4. *Patients with more than two clinical cardiac risk factors and poor functional capacity (4 or fewer METs) should undergo imaging stress-testing before pelvic exenteration.* Class I/level C (81 per cent)
5. *Formal assessment by a cardiologist is not required routinely for patients requiring pelvic exenteration.* Class I/level C (86 per cent)

#### Low consensus statement

1. *Patients requiring pelvic exenteration do not need to be admitted routinely the night before surgery, unless specifically indicated (for example, for optimization).* Class IIb/level C (60 per cent)

### Section 2: Anaesthetic considerations

Pelvic exenteration is typically performed through an open abdominal approach, although recent advancements in laparoscopic techniques and robotic surgery have enabled successful minimally invasive pelvic exenterative procedures to be performed. In view of the radical and complex nature of the surgery and the expected extended duration required, general anaesthesia is mandatory, whereas combined epidural and general anaesthesia may be considered for additional postoperative pain control^12^. Concomitant neuraxial blockade with epidural or spinal anaesthesia further induces sympathetic blockade, thereby attenuating the stress response associated with surgery. There is evidence suggesting improved survival and a reduced incidence of major cardiopulmonary and thromboembolic problems, with more distinct benefits appreciated among patients at high risk of developing complications^13–17^.

Although the administration of an inhaled volatile anaesthetic is the commonest technique used for maintenance of anaesthesia in the UK and Ireland^18^, there has been emerging evidence indicating that volative anaesthetics are proinflammatory and consequently affect immune processes including the immune response to surgery, hence increasing the risk of postoperative cancer recurrence and negatively impacting survival^19–21^. In total intravenous anaesthesia (TIVA), the maintenance of general anaesthesia is by intravenous infusion, with advantages such as reduced risk of postoperative nausea, rapid recovery of consciousness, and possibly superior oncological outcomes^22–24^. Nevertheless, potential problems that may be encountered in TIVA include failure in intended drug delivery, underdosing resulting in accidental awareness, and overdosing. Furthermore, inconsistent and inadequate training with this anaesthetic technique is an exacerbating factor, limiting its adoption among anaesthetists^18^. Hence, there has been no consensus on the suitability of TIVA in pelvic exenterative procedures, or on the intraoperative use of processed electroencephalographic monitoring to prevent awareness and overdosing of anaesthetic drugs.

Selection of the most suitable haemodynamic and respiratory intraoperative monitoring modalities, and appropriate integration of these various parameters can ensure a more comprehensive understanding of the haemodynamic status of the patient. This provides a broader clinical overview to facilitate intraoperative decision-making, hence avoiding under- or over-resuscitation, which are both detrimental^25^^,^^26^. In the context of pelvic exenteration, it may not be possible to measure urine output meaningfully in some patients where the bladder and/or ureter(s) are resected. Therefore, besides continuous invasive BP measurement, intra-arterial line placement allows for the analysis of arterial blood gases and blood tests at regular time intervals throughout the case. Where haemodynamic instability can be expected, the use of vasopressors, inotropic agents, or large-volume resuscitation with fluids and blood products mandates the placement of a central venous line and/or large-bore vascular catheter.

The need for a dedicated anaesthetic team for exenterative procedures cannot be overemphasized, especially for high-risk patients undergoing pelvic exenteration. It allows for centralization of expertise and clinical experience, and ensures smooth coordinated workflow, which is especially critical in resuscitative scenarios. In anticipated high-risk exenterative cases with a significant risk of clinical instability during the prolonged duration of the procedure, further assistance from another anaesthetic consultant or senior colleague should be considered.

#### Consensus recommendations

1. *Arterial line placement for invasive monitoring during pelvic exenteration.* Class I/level C (100 per cent)
2. *Large-bore vascular catheter should be placed at the discretion of the anaesthetist.* Class I/level C (97 per cent)
3. *Arterial blood gas and haemoglobin levels should be monitored regularly during pelvic exenteration.* Class I/level C (100 and 93 per cent respectively)
4. *The plasma lactate level should be kept below 1 mmol/l or as low as possible to ensure optimal perfusion and aerobic metabolism.* Class I/level C (80 per cent)
5. *If the pelvic exenteration is anticipated to be high-risk and expected duration is more than 12–18 h, another anaesthetic consultant or colleague should be readily available to assist throughout the procedure.* Class I/level C (89 per cent)
6. *If possible, there should be a dedicated anaesthesia team of nurses or operating department practitioners who have been specifically trained to work with the anaesthetist during pelvic exenteration.* Class I/level C (86 per cent)

##### Low consensus statements

1. *Central venous line placement is useful in most pelvic exenteration procedures.* Class IIa/level C (78 per cent)
2. *TIVA is not suitable for pelvic exenteration.* Class IIb/level C (53 per cent)
3. *Electroencephalographic/bispectral index monitoring is useful to prevent awareness and overdosing of anaesthetic drugs during pelvic exenteration.* Class IIb/level C (46 per cent)
4. *Non-invasive cardiac output (NICO) monitoring should be used during pelvic exenteration to monitor preload and obtain optimal cardiac output.* Class IIb/level C (61 per cent)

### Section 3: Perioperative management

The universal implementation of the WHO Surgical Checklist has been shown that safety is beneficial for teamwork and communication in the operating theatre, thereby improving patient outcomes^27^. Subsequently, the adoption of regular preoperative team briefing demonstrated a significant decrease in work-flow disruptions during surgery^28^. For more complex operations such as pelvic exenteration, which require multidisciplinary and interprofessional coordination of care over an extended duration of surgery, a combined and structured team brief is essential. This ensures a platform for open discussion of the case, facilitates collective efforts to address anticipated issues, and allows ample time for preparation or rectification if required.

Although it is widely accepted that patients undergoing major abdominopelvic surgery are at increased risk of developing postoperative venous thromboembolic (VTE) complications^29^^,^^30^, the role of perioperative pharmacological thromboembolic prophylaxis remains debatable, especially among Asian surgical patients who are known to have a significantly lower risk of developing VTE complications^31^. No consensus was obtained regarding the perioperative administration of low molecular weight heparin at prophylactic dose within 24 h, whether before or after pelvic exenterative surgery. Routine antibiotic prophylaxis should have aerobic and anaerobic coverage, guided by the allergy status of the patient.

There is substantial variation in the types of operating table and mattress currently used by different centres worldwide. An operating table suitably equipped with full mobility and flexibility, while retaining good stability, should be used. The table should be able to cope with Lloyd-Davies, lithotomy, steep Trendelenburg and supine positions, and allow for very low table height should the need arise.

The operating table mattress may be used in conjunction with a non-slip warming gel pad, combining their properties to achieve the three aims of providing adequate support and comfort, helping to maintain positional stability, and preserving normothermia for prolonged periods. Various warming systems are available commercially to prevent inadvertent hypothermia during the procedure, which can potentially contribute to coagulopathy, immune function suppression, cardiac and infective complications. In contrast, should the core body temperature of the patient be increased during surgery owing to an inflammatory response from the extirpative phase of the surgery, consideration may be given for the warming adjuncts to be stepped down or switched off.

Patient manoeuvring and extended duration in a fixed position during surgery can potentially result in injuries sustained while under anaesthesia. All patient positions can result in large degrees of pressure concentrated disproportionately on small, specific parts of the body surface, resulting in reduced perfusion and tissue ischaemia, consequently leading to the development of pressure injuries. The spectrum of severity can range from erythema on intact skin or partial-thickness skin loss, to tissue destruction involving subcutaneous fat, muscle and bone. Cardiovascular disease, respiratory disease, diabetes mellitus, anaemia and duration of surgery are significant perioperative risk factors for the development of pressure injury as a result of surgery under general anaesthesia^32^. Peripheral nerve injury can occur where the nerve is subjected to stretch, compression, ischaemia, trauma, or a combination of these factors^33^, resulting in pressure-related neuropathy, particularly among thin patients who have undergone previous neoadjuvant chemoradiotherapy.

In the Lloyd-Davies and modified Lloyd-Davies positions, elevation of the patient’s legs at a level above the heart causes a decrease in perfusion pressure, tissue ischaemia, and hypoxic disruption of capillary endothelium, leading to interstitial oedema and a rise in leg intracompartmental pressure^34^^,^^35^. This can be further exacerbated by the Trendelenburg position for an extended duration during surgery. Recent guidelines^36^ from a multidisciplinary collaboration of colorectal, vascular and orthopaedic surgeons, acting on behalf of their specialty associations in the UK and Ireland, have similarly recommended that the patient’s legs should be kept at a level below the heart for the maximum duration possible during pelvic surgery. When leg elevation is required, the continuous duration of elevation should be limited to 4 h^36^.

Special consideration should be given to the prone position, which is frequently associated with cardiopulmonary issues. Abdominal compression and increased intra-abdominal pressure cause direct pressure on inferior vena cava, hence venous pooling, decreased venous return and reduced cardiac output ensue^37^. Increased thoracic pressure and decreased respiratory compliance in the prone position leads to an increase in peak airway pressure, thereby also decreasing venous return and cardiac output^38^.

Besides physiological derangements, the prone position is also frequently implicated with position-associated injuries. Sufficient staffing and due care must be ensured during the manoeuvring of the patient from supine to prone position. Appropriate padding equipment is necessary to offset excessive pressure from the face, particularly the nose and eyes. Hyperflexion and hyperextension of the cervical spine should be avoided. Careful positioning of the arms can help to prevent brachial and/or ulnar neuropathy secondary to plexus and/nerve impingement. Finally, the pressure points and skin must be checked before proceeding with the next phase of the exenterative surgery.

#### Consensus recommendations

1. *For every patient requiring pelvic exenteration, the MDT briefing should be held routinely before the induction of anaesthesia. This ensures open communication and sharing of vital surgical, anaesthetic, nursing and logistical concerns, thereby enabling coordinated efforts to address any conflicting considerations, and discussion of strategies to deal with possible difficulties.* Class I/level C (94 per cent)
2. *Mechanical calf compression and thromboembolic deterrent stockings should be used regularly during pelvic exenteration.* Class I/level C (86 per cent)
3. *Careful pressure point protection padding, particularly at the sacrum, should be used to prevent pressure ulcers.* Class I/level C (89 per cent)
4. *To prevent leg compartment syndrome during pelvic exenteration, careful vigilance on leg positioning is crucial.* Class I/level C (87 per cent)
5. *Should leg elevation be required during a long pelvic exenterative procedure, the legs should be brought down every 2–4 h, where possible.* Class I/level C (84 per cent)
6. *Patients undergoing pelvic exenteration may need to be placed in the prone position for surgical access or myocutaneous flap reconstruction.* Class I/level C (85 per cent)
7. *Patients undergoing pelvic exenteration usually remain sufficiently haemodynamically stable to prone, if required during the procedure. I/C (92 per cent).*

#### Low consensus statement

1. *Low molecular weight heparin should be given routinely within 24 h of the perioperative period.* Class IIa/level C (71 per cent)

### Section 4: Anticipating possible massive haemorrhage

Depending on the tumour location and structures involved by tumour and/or fibrosis, an exenterative procedure may entail the resection of pelvic organs, such as the bladder, prostate, seminal vesicles, urethra, vagina and uterus, and/or part of the sacrum. In addition, lateral pelvic side-wall and iliac vessel dissection may be required^39^^,^^40^.

Although exenterative surgeons acknowledge that significant bleeding can arise from the iliac vasculature and the sacral venous plexus during pelvic exenteration, the risk of bleeding can differ widely among patients. This is due largely to the heterogeneity existing in individual patient anatomy, type of exenterative procedure required, and the presence of fibrosis and adhesions from pelvic irradiation or previous surgery. The prospect of sudden massive haemorrhage during pelvic exenteration, where rapid and aggressive resuscitation with intravenous fluids and blood products is necessary to avoid haemodynamic instability and the downstream cascade of complications, presents a major challenge to the anaesthetist^41^.

Preoperative haemoglobin optimization is imperative; however, owing to limited evidence regarding the ideal haemoglobin level before surgery and lack of consensus on the most efficacious strategy in treating varying degrees and different causes of anaemia^42^, no standardized protocol has been implemented widely among patients undergoing pelvic exenteration. Intravenous iron infusion replenishes iron stores, allowing for a rapid and more complete haematological response to mitigate operative blood loss, reducing the risk of allogeneic blood transfusion and perioperative morbidity, which can negatively impact the oncological outcome.

The use of an antifibrinolytic agent such as tranexamic acid (TXA) at the time of surgery may have a substantial impact in mitigating the risk and severity of intraoperative bleeding^43^^,^^44^. Dosing regimens vary among the various guidelines, but the administration of 1 g TXA to all patients undergoing surgery where significant blood loss is likely or possible can be considered, with no evidence to support the use of high doses^45–47^.

Every institution should have a massive transfusion protocol in place that is audited regularly to ensure logistical readiness when needed. During active massive surgical haemorrhage, transfusion should ideally be guided by the evolving clinical circumstances and point-of-care testing of blood, such as thromboelastography (TEG^®^: Haemonetics, Boston, MA, USA). With a more rapid turnaround time, this provides a more accurate representation of the dynamic coagulation profile, enabling targeted blood component therapy and optimized component use.

#### Consensus recommendations

1. *Patients undergoing pelvic exenteration may be transfused a variable quantity of blood.* Class I/level C (97 per cent)
2. *There should be 2 units (or more) of blood cross-matched for each patient.* Class I/level C (84 per cent)
3. *TXA can be given, if required, during surgery.* Class I/level C (81 per cent)
4. *Patients undergoing pelvic exenteration may haemorrhage very suddenly and quickly.* Class I/level C (91 per cent)
5. *There is a risk of major haemorrhage in exenterative procedures.* Class I/level C (97 per cent)
6. *When exenterative procedures involve undergoing major haemorrhage, thromboelastography, if available, is useful to guide the administration of clotting products.* Class I/level C (84 per cent)
7. *Blood products and reconstituted blood products should be freely available for immediate use if demanded by the anaesthetist to eradicate time delays.* Class I/level C (85 per cent)

#### Low consensus statement

1. *For exenterative procedures involving massive transfusion (according to individual hospital protocol), haematologist guidance is often not required.* Class IIa/level C (76 per cent)

### Section 5: Stress response and postoperative critical care

The initial stress response to surgery is thought to be beneficial, but the excessive and prolonged activation of the inflammatory and immunological components of the response has been associated with adverse postoperative sequelae^48^^,^^49^. Despite the original protective nature of the response, an overwhelming cytokine storm, once elicited, can result in a massive inflammatory cascade and the systemic inflammatory response syndrome (SIRS), leading to secondary multiple organ dysfunction and death.

Although the precise risk of developing SIRS remains unknown, patients undergoing pelvic exenteration invariably develop a certain degree of inflammatory response, particularly as extensive surgery in patients with cancer can cause the dysregulated release of proinflammatory cytokines by activated leucocytes, fibroblasts and endothelial cells. In patients undergoing pelvic exenteration, SIRS may be exacerbated by other factors, including coexisting infections and impaired nutritional status. Furthermore, the greater likelihood of intraoperative blood transfusion required in exenterative surgery may predispose the patient to a more exaggerated immune response. This is evidenced by retrospective data indicating a significant association between perioperative blood transfusion and the postoperative systemic inflammatory response^50^^,^^51^.

The goals of perioperative anaesthetic management should therefore include minimizing the stress response, through neuraxial blockade for example. In addition, early identification and timely recognition of SIRS among patients undergoing pelvic exenteration can allow appropriate supportive management to be instituted to prevent further end-organ damage. This may consist of the use of vasopressors and inotropic agents, mechanical ventilatory support, and a period of treatment in the critical care unit (CCU). Typically, CCU admission is necessary after exenterative surgery^52^, facilitating continuous clinical monitoring, enhanced nursing care, and close critical care management in the early postoperative phase.

Fluid management is an essential component of the postoperative critical care of the patient undergoing pelvic exenteration. The evidence comparing restrictive fluid strategy with goal-directed fluid therapy (GDFT) in patients undergoing major non-cardiac surgery remains inconclusive. In a recent systematic review^53^, the data were derived from studies consisting of mainly low-risk patients undergoing abdominal surgery. However, there has been considerable evidence underlining the advantages of GDFT in reducing morbidity and mortality in the high-risk surgical patient population^54–56^. GDFT aims to optimize the haemodynamic status in order to achieve effective oxygen delivery to tissues, through judicious fluid administration and inotrope use, as guided by the monitoring of haemodynamic variables such as cardiac output, cardiac index and stroke volume.

Cardiac output monitoring has traditionally been performed with the pulmonary artery thermodilution technique using the pulmonary artery catheter (PAC) and transpulmonary thermodilution. With the emergence of less invasive and non-invasive technologies for cardiac output monitoring, it has become used less commonly in the setting of perioperative and intensive care. Nevertheless, inconsistency and variability between the commercially available NICO monitoring technologies^57^ have limited their reliability and wider clinical applicability. Therefore, no consensus was reached regarding the routine use of NICO monitoring during surgery (Section 2) or for postoperative care following pelvic exenterative procedures.

#### Consensus recommendations

1. *Patients undergoing pelvic exenteration may develop a large systemic inflammatory response (SIR), which may result in instability.* Class I/level C (95 per cent)
2. *Should the patient develop SIRS, it is likely that they will require inotropy.* Class I/level C (89 per cent)
3. *If clinically required, the recommended inotrope to use initially is noradrenaline.* Class I/level C (85 per cent)
4. *Patients undergoing pelvic exenteration who develop major SIRS or instability on inotropes should be transferred to the CCU for stabilization before extubation.* Class I/level C (94 per cent)
5. *CCU admission is usually required in the perioperative period for invasive monitoring and/or organ support in patients undergoing pelvic exenteration.* Class I/level C (81 per cent)
6. *GDFT is the most appropriate fluid strategy after surgery.* Class I/level C (85 per cent)

#### Low consensus statements

1. *The degree of SIR encountered is not predictable from the preoperative assessment.* Class IIb/level C (61 per cent)
2. *In patients undergoing pelvic exenteration with SIRS, large volumes of fluid can be given to maintain preload and adequate circulation and metabolism, as measured by NICO and arterial blood gases.* Class IIb/level C (59 per cent)
3. *Patients undergoing pelvic exenteration should be adequately filled*  *after surgery to maintain optimal perfusion with lactate less than 1 mmol/l.* Class IIb/level C (67 per cent)
4. *Based on differences in individual patient profile and physiology, and variability in the nature and extent of exenterative procedures, it is not possible to have a standardized approach to fluid management or inotropic support.* Class IIa/level C (73 per cent)

### Section 6: Pain management

Postoperative pain management should be a continuation of intraoperative analgesia, utilizing a multimodal opioid-sparing approach where possible. In the current era of enhanced recovery after colorectal resection, minimization of systemic opioid use is associated with earlier recovery of gastrointestinal function, shorter duration of hospital stay, and fewer postoperative complications^58–60^. Numerous adjunct local and regional analgesic techniques, including epidural analgesia, spinal analgesia, intravenous lidocaine infusion, transversus abdominis plane block, and continuous wound infusion of local anaesthetics via a catheter^61^, are effective postoperative pain control modalities, each with their risks and benefits.

For instance, epidural analgesia is a consistently effective pain control technique after major colorectal resection. It can help to alleviate the sympathetic stress response through neuraxial blockade. Additionally, when used in conjunction with a general anaesthetic and maintained for at least 24 h after surgery, epidural analgesia decreases postoperative mortality, probably due to concomitant reductions in major cardiopulmonary and gastrointestinal complications^61^^,^^62^. However, epidural analgesia can potentially cause hypotension^63^, pruritus, incomplete block and, rarely, severe neurological complications.

Satisfactory postoperative analgesia for the patient who has undergone pelvic exenteration can be a multifaceted anaesthetic challenge, regardless of the analgesic technique(s) chosen. Although individual evaluation and comparative analyses of these techniques are beyond the scope of this consensus statement, there are limitations inherent to every technique due to side-effects, variable pharmacokinetics and pharmacodynamics of different drugs, and technical problems with drug administration. Moreover, apart from the interindividual differences in pain threshold, the pain intensity is variable based on the different surgical approaches, size of incision(s), and type of exenterative and reconstructive procedures performed.

Similarly, the expected postoperative recovery milestones differ widely even among patients undergoing pelvic exenteration; therefore, it may be necessary for the overall postoperative care plan to be individualized according to the considerations and issues unique to each patient. Furthermore, although it is unrealistic for entire enhanced recovery protocols following routine colorectal resection to be applied to patients undergoing pelvic exenteration, certain principles pertaining to medical and nutritional optimization, pain control, mobilization, and exercise interventions can be integrated into the recovery plan after exenterative procedures.

#### Consensus recommendations

1. *Effective regional techniques (such as epidural, spinal anaesthesia, regional blocks) or intravenous lidocaine are superior to intravenous opiates.* Class I/level A (84 per cent)
2. *In reducing postoperative intravenous opioid use, epidural is useful in pelvic exenterative procedures.* Class I/level A (86 per cent)
3. *Patients undergoing pelvic exenteration should have a careful postoperative pain management protocol in place to ensure good regional anaesthesia is maintained with practitioners who can implement changes to epidural pain management.* Class I/level C (89 per cent)

#### Low consensus statements

1. *Epidurals can have a positive effect on microcirculation and optimal perfusion.* Class IIb/level C (51 per cent)
2. *The epidural catheter should be tunnelled so that it can potentially last 10 days.* Class IIb/level C (54 per cent)

## Discussion

There is a need for further studies in various domains of the present perioperative and anaesthetic management framework. First, risk stratification of patients requiring pelvic exenteration can help to define the high-risk population that may benefit from more rigorous preoperative assessment and intensive perioperative management such as invasive cardiac output monitoring during and/or after the surgical procedure. Moreover, given the diverse clinical practice and protocols among the exenterative centres, this consensus can serve as a platform for more streamlined perioperative management and the development of coordinated care pathways for patients undergoing pelvic exenteration. Second, the evidence on the optimal fluid management strategy, especially for high-risk patients undergoing major abdominal surgery such as pelvic exenteration, is lacking at present. Furthermore, there is considerable heterogeneity among different patient profiles, as well as the different types of exenterative procedure performed with varying extents of blood loss. Lastly, with the emerging adoption of enhanced recovery protocols worldwide, future data and guidelines may better ascertain the applicability of specific principles such as perioperative nutritional optimization and exercise interventions, and evaluate the extent of beneficial effect among patients requiring pelvic exenteration. Although some tenets of enhanced recovery have been incorporated in the present consensus, such as thromboembolic prophylaxis, multimodal analgesia and avoidance of opiates, there are other aspects for which consensus could not be achieved. For instance, intraoperative anaesthetic protocols and fluid management, and the use of minimally invasive surgical approaches^64^, saw significant differences in practices across the various exenterative centres.

*Table 3* provides a summary of consensus statements.

### Collaborators

Members of the PELVEX Collaborative: A. Y. Chok, A. Oliver, S. Rasheed, E. J. Tan, M. E. Kelly, A. G. J. Aalbers, N. Abdul Aziz, N. Abecasis, M. Abraham-Nordling, T. Akiyoshi, W. Alberda, M. Albert, M. Andric, E. Angenete, A. Antoniou, R. Auer, K. K. Austin, O. Aziz, R. P. Baker, M. Bali, G. Baseckas, B. Bebington, M. Bedford, B. K. Bednarski, G. L. Beets, P. L. Berg, J. Beynon, S. Biondo, K. Boyle, L. Bordeianou, A. B. Bremers, M. Brunner, P. Buchwald, A. Bui, A. Burgess, J. W. A. Burger, D. Burling, E. Burns, N. Campain, S. Carvalhal, L. Castro, A. Caycedo-Marulanda, K. K. L. Chan, G. J. Chang, M. H. Chew, P. Chong, H. K. Christensen, H. Clouston, M. Codd, D. Collins, A. J. Colquhoun, A. Corr, M. Coscia, P. E. Coyne, B. Creavin, R. S. Croner, L. Damjanovic, I. R. Daniels, M. Davies, R. J. Davies, C. P. Delaney, J. H. W. de Wilt, Q. Denost, C. Deutsch, D. Dietz, S. Domingo, E. J. Dozois, M. Duff, T. Eglinton, J. M. Enrique-Navascues, E. Espin-Basany, M. D. Evans, N. S. Fearnhead, K. Flatmark, F. Fleming, F. A. Frizelle, M. A. Gallego, E. Garcia-Granero, J. L. Garcia-Sabrido, L. Gentilini, M. L. George, V. George, L. Ghouti, F. Giner, N. Ginther, R. Glynn, T. Golda, B. Griffiths, D. A. Harris, J. A. W. Hagemans, V. Hanchanale, D. P. Harji, R. M. Helewa, G. Hellawell, A. G. Heriot, D. Hochman, W. Hohenberger, T. Holm, A. Holmström, R. Hompes, J. T. Jenkins, S. Kaffenberger, G. V. Kandaswamy, S. Kapur, Y. Kanemitsu, S. R. Kelley, D. S. Keller, M. S. Khan, H. Kim, H. J. Kim, C. E. Koh, N. F. M. Kok, R. Kokelaar, C. Kontovounisios, H. Ø. Kristensen, H. M. Kroon, M. Kusters, V. Lago, S. G. Larsen, D. W. Larson, W. L. Law, S. Laurberg, P. J. Lee, M. Limbert, M. L. Lydrup, A. Lyons, A. C. Lynch, C. Mantyh, K. L. Mathis, C. F. S. Margues, A. Martling, W. J. H. J. Meijerink, S. Merkel, A. M. Mehta, D. R. McArthur, F. D. McDermott, J. S. McGrath, S. Malde, A. Mirnezami, J. R. T. Monson, J. R. Morton, T. G. Mullaney, I. Negoi, J. W. M. Neto, B. Nguyen, M. B. Nielsen, G. A. P. Nieuwenhuijzen, P. J. Nilsson, S. T. O’Dwyer, G. Palmer, E. Pappou, J. Park, D. Patsouras, G. Pellino, A. C. Peterson, G. Poggioli, D. Proud, M. Quinn, A. Quyn, R. W. Radwan, P. C. Rasmussen, E. Rausa, S. E. Regenbogen, A. Renehan, R. Rocha, M. Rochester, J. Rohila, J. Rothbarth, M. Rottoli, C. Roxburgh, H. J. T. Rutten, É. J. Ryan, B. Safar, P. M. Sagar, A. Sahai, A. Saklani, T. Sammour, R. Sayyed, A. M. P. Schizas, E. Schwarzkopf, V. Scripcariu, C. Selvasekar, I. Shaikh, D. Shida, A. Simpson, N. J. Smart, P. Smart, J. J. Smith, A. M. Solbakken, M. J. Solomon, M. M. Sørensen, S. R. Steele, D. Steffens, K. Stitzenberg, L. Stocchi, N. A. Stylianides, T. Swartling, H. Sumrien, P. A. Sutton, T. Swartking, C. Taylor, J. Teras, R. Thurairaja, E. L. Toh, P. Tsarkov, Y. Tsukada, S. Tsukamoto, J. J. Tuech, W. H. Turner, J. B. Tuynman, G. H. van Ramshorst, D. van Zoggel, W. Vasquez-Jimenez, C. Verhoef, G. Vizzielli, E. L. K. Voogt, K. Uehara, C. Wakeman, S. Warrier, H. H. Wasmuth, K. Weber, M. R. Weiser, J. M. D. Wheeler, J. Wild, M. Wilson, A. Wolthuis, H. Yano, B. Yip, J. Yip, R. N. Yoo, D. C. Winter, P. P. Tekkis.
